# Efficient Extraction of Anti-Inflammatory Active Ingredients from *Schefflera octophylla* Leaves Using Ionic Liquid-Based Ultrasonic-Assisted Extraction Coupled with HPLC

**DOI:** 10.3390/molecules24162942

**Published:** 2019-08-14

**Authors:** Xuqiang Liu, Yun Niu, Jieqing Liu, Mengjun Shi, Ruian Xu, Wenyi Kang

**Affiliations:** 1 Engineering Research center of Molecular Medicine, Ministry of Education, Xiamen 361021, China; 2 National R & D Center for Edible Fungus Processing Technology, Henan University, Kaifeng 475004, China; 3 School of Biomedical Sciences and School of Medicine, Huaqiao University, Xiamen 361021, China; 4 Joint International Research Laboratory of Food & Medicine Resource Function, Kaifeng 475004, China

**Keywords:** *Schefflera octophylla* (Lour.) Harms, ionic liquid, response surface method

## Abstract

*Schefflera octophylla* (Lour.) Harms, a kind of traditional Chinese medicine (TCM), is commonly used for anti-inflammatory, analgesic, rheumatism, fever, and hemostasis therapy. In our previous studies, two major triterpenoids were isolated and identified from leaves of *S. octophylla*, and evaluated for their inhibitory effects on lipopolysaccharide (LPS)-induced nitric oxide production in RAW264.7 cells; both of them displayed significant anti-inflammatory activities at their noncytotoxic concentrations. Therefore, it is very useful to establish an efficient and green extraction method to isolated the two major triterpenoids from leaves of *S. octophylla*. In this paper, ionic liquid based ultrasonic-assisted extraction (ILUAE) was successfully applied to extract the two major triterpenoids from leaves of *S. octophylla*. Four single factors (ionic liquids (ILs) concentration, solid–liquid ratio, centrifugal speed, mesh number), with a greater impact on extraction rate, were selected from a variety of influencing factors, and the optimal conditions were obtained by Box–Behnken response surface methodology (RSM). Under optimal conditions, the total extraction yield and extraction rate of two triterpenoids were 288.03 mg/g and 28.80%, respectively, which was 6.80% higher than that of 70% Ethanol (220 mg/g and 22%, respectively).

## 1. Introduction

**Figure 1:**
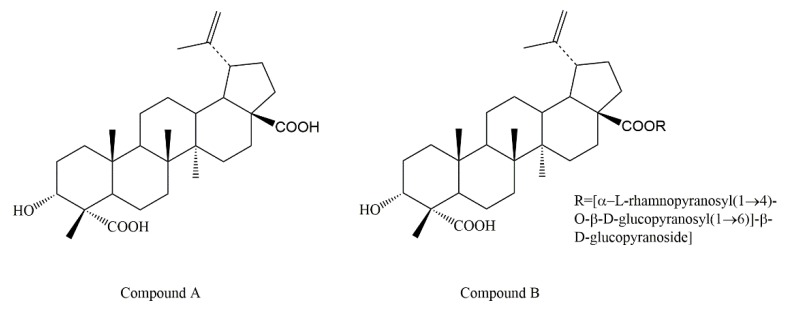
Chemical structures of compounds **A** and **B**.

*Schefflera octophylla* (Lour.) Harms, belonging to the Araliaceae family, is distributed mainly in Guangxi, Guangdong, Fujian, and Yunnan provinces in China. It is widely used as a folk medicine for the rheumatic pain, traumatic pain, and sore throat [1]. In recent years, pharmacological studies have shown that *S. octophylla* has a variety of biological activities, such as antitumor [2], antivirus [3], and anti-inflammatory [4,5] effects. Previous phytochemical investigations have also revealed that triterpenoids were the main components and more than 40 triterpenoids were isolated from this plant [6,7,8,9,10,11,12,13]. Among all these triterpenoids, 3α-hydroxy-lup-20(29)-ene-23,28-dioic acid (compound **A**) and 3α-hydroxy-lup-20(29)-ene-23,28-dioic acid 28-*O*-[α-l-rhamnopyranosyl(1→4)-*O*-β-d-glucopyranosyl(1→6)]-β-d-glu-copyranoside (compound **B**) (Figure 1) were the main components of *S. octophylla*. Moreover, our previous unpublished study indicates that compound **A** and compound **B** had significant anti-inflammatory activity, indicating that they may be the material basis of the anti-inflammatory activity. Therefore, it is meaningful to improve the extraction rate of compound **A** and compound **B** from leaves of *S. octophylla.*

There are many traditional methods in the extraction of active ingredients from TCM, such as immersion method, percolation method, frying method, and reflux extraction method. However, traditional extraction methods have many drawbacks such as being time-consuming, inefficient, and pollution to the environment. In recent years, ionic liquids (ILs), as a kind of novel solvents in the extraction of bioactive compounds, such as alkaloids [14], flavonoids [15,16], glycosides [17], and terpenoids [18], have been successfully applied. The extraction of bioactive components from plants using ILs is promising as a green method, which could reduce the pollution of environment and improve the selectivity and extraction yields of target analytes. ILs as a new type of green organic solvent had good thermal and chemical stability, viscosity range, adjustable, and good solubility characteristics [19]. To our best knowledge, some assisted technologies could be coupled with ILs, such as microwave-assisted extraction (MAE) [20], ultrasonic-assisted extraction (UAE) [21], and ionic liquid-based negative pressure cavitation-assisted extraction (IL-NPCE) [22]. All these combined technologies exhibited marked improvements in the fields of extraction. Among them, ultrasound-assisted extraction has been successfully used for the extraction of the bioactive constituents from the plant materials [14], which has many advantages, such as high reproducibility, reduction in extraction time, minimum solvent consumption and minimum energy output.

Response surface methodology (RSM), widely used for optimization of the extraction process of the bioactive ingredients, is an effective statistical technique for optimizing complex processes. It also allows more efficient and easier arrangement and interpretation of experiments compared with other methods [20].

At present, the ionic liquids-based ultrasonic assisted extraction (IL-UAE) method has been proven as a more effective method in extracting various compounds from natural plants [14,15,16,17,18]. However, there is still no related report about the ionic liquids-based ultrasonic assisted extraction (IL-UAE) of compound **A** and compound **B** from leaves of *S. octophylla*. Thus, our study aimed to develop a rapid and efficient ionic liquids-based ultrasonic assisted extraction method (IL-UAE) combined with high performance liquid chromatography (HPLC) to determine the anti-inflammatory active ingredients (compounds **A** and **B**). In this paper, the effects of seven single factors on extraction yield of target analytes were investigated, and then four single factors (concentration of ILs, solid–liquid ratio, centrifugal speed, and crushed mesh), with a greater impact on the extraction rate of the target analytes, were selected for further experimentation. Although the extraction yield could be affected by many factors and interactions, the response surface methodology (RSM) has been proven to be an efficient way to select optimum conditions [23]. In order to determine the optimal conditions, response surface method was applied to screen the single factor using Design-Expert 8.06 statistical analysis software. 

## 2. Results and Discussion

### 2.1. Effects of Compound **A** and Compound **B** on Cells Viability

**Figure 2:**
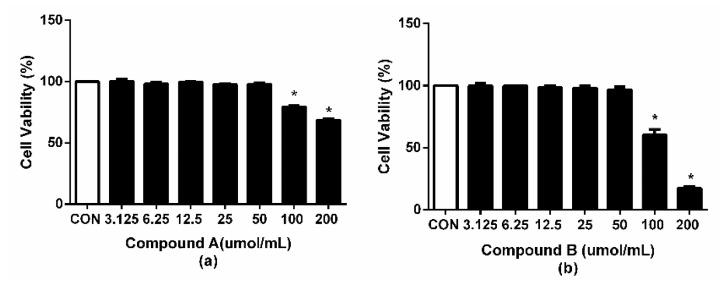
Cytotoxicity of compound **A** (**a**) and compound **B** (**b**) in RAW264.7 cells, which were treated with different concentrations of compound **A** and **B** for 24 h. * *p* < 0.05 compared with the control group.

A preliminary experiment was established, wherein compound **A** and compound **B** treatment was performed for 24 h, and RAW264.7 cell viability was decreased at 100 µmol/mL (*p* < 0.01) and 50 µmol/mL (*p* < 0.01) (Figure 2a,b). Thus, the following experiments were performed within 100 µmol/mL of compound **A** and 50 µmol/mL of compound **B**. The results showed that the cell viability between compound **A** and compound **B** had some differences, which might be related to the three monosaccharides in c-28 of compound **B**.

### 2.2. Effects of Compound **A** and Compound **B** on NO Production in Lipopolysaccharide (LPS)-Induced RAW 264.7 Macrophages

**Figure 3:**
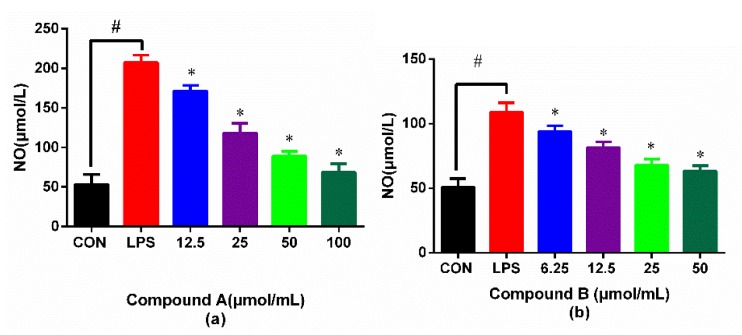
Inhibitory effects of compound **A** (**a**) and compound **B** (**b**) on lipopolysaccharide (LPS)-induced NO production in RAW264.7 macrophages. # *p* < 0.05 compared with the control group; * *p* < 0.05 compared with LPS group.

As shown in Figure 3a,b, the level of NO in the lipopolysaccharide (LPS) group was significantly higher than that of the CON group (*p* < 0.05), which indicated that the inflammatory cell model was successfully constructed. When the concentration of compound **A** and **B** increased, the inhibition of NO releasing was significantly elevated, and presented a tendency of a concentration-dependent manner. The results implied that compound **A** and compound **B** both had significant anti-inflammatory activity. However, the anti-inflammatory effects of the two compounds had some differences, which might be related to their structural differences. Compound **B** has three monosaccharides in c-28 compared with compound **A**, which might have an effect on its anti-inflammatory activity.

### 2.3. Selection of ILs

**Figure 4:**
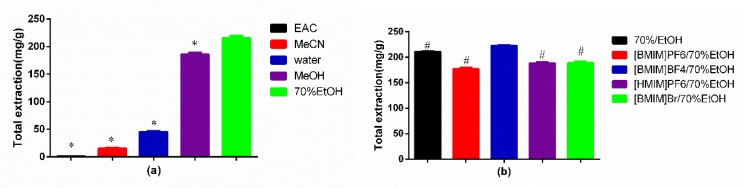
(**a**) Effects of five conventional extraction agents on extraction yields of target analytes; (**b**) effects of four kinds of ILs with 70%EtOH on extraction yields of target analytes. * *p* < 0.05, compared with 70%EtOH group; # *p* < 0.05, compared with [BMIM]BF_4_/70%EtOH group.

Generally, the selection of appropriate extraction solvent is the first step of the whole analysis experiment. There were five conventional extraction solvents (ethyl acetate (EAC), acetonitrile (MeCN), water, methanol (MeOH), and 70% ethanol (70%EtOH)), which were used to extract the active ingredients from TCM. According to their different polarities and penetrating forces, chemical components suitable for extraction are not the same. In this paper, the data are shown in Figure 4a, which state clearly that the extraction yield with 70% ethanol (70%EtOH) was higher than those of other solvents (*p* < 0.05). Thus, 70%EtOH was the most suitable extraction agent for compounds **A** and **B**.

As ionic liquid is the core factor of this experiment, it is extremely important to select the suitable ionic liquid type. In our previous study [24,25,26,27,28,29], four imidazole ILs ([BMIM]BF_4_ (1-Butyl-3-methylimidazolium tetrafluoroborate), [BMIM]Br(1-butyl-3-methylimidazolium bromide), [BMIM]PF_6_(1-butyl-3-methylimidazolium hexafluorophosphate), and [HMIM]PF_6_(1-Hexyl-3- methylimidazolium hexafluorophosphate)) could obviously improve the extraction yield. It was probably because of its stronger multi-interactions including π–π, ionic/charge–charge, and hydrogen bonding with those compounds [30]. The extraction yield of four kinds of ILs with 70% EtOH (the concentration of ILs: 0.6 mol/L) on target analytes is shown in Figure 4b, which displayed that the extraction rate of [BMIM]BF_4_/70%EtOH was obviously higher than those of other kinds of ILs with 70% EtOH (*p* < 0.05). In addition, the extraction rate of [BMIM]BF_4_/70%EtOH was also higher than that of 70% EtOH (*p* < 0.05). Thus, [BMIM]BF_4_ was selected for further study.

### 2.4. Single Factor Experiments

#### 2.4.1. Selection of the Concentrations of ILs 

**Figure 5:**
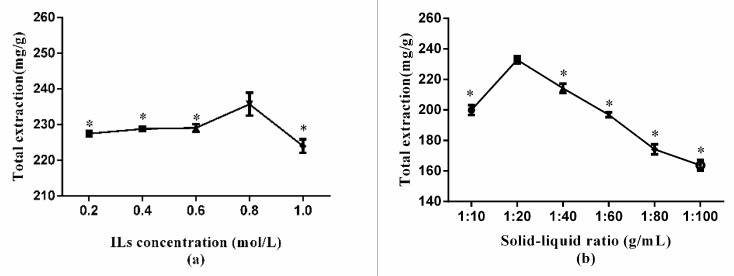

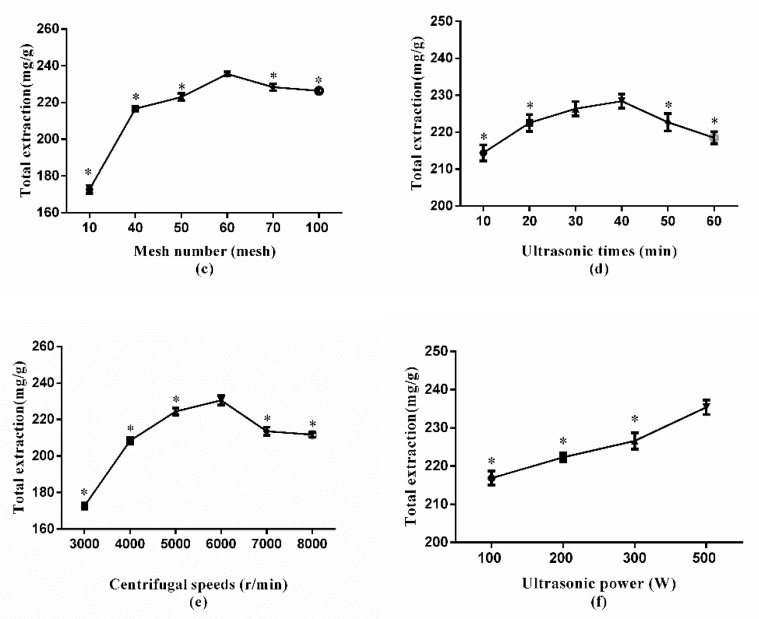
Extraction yields of ionic liquids-based ultrasonic assisted extraction (IL-UAE) affected by (**a**) ILs concentration temperature, when compared with 0.8 group: * *p* < 0.05; (**b**) solid–liquid ratio, compared with 1:20 group: * *p* < 0.05; (**c**) mesh number, compared with 60 group: * *p* < 0.05; (**d**) ultrasonic time, compared with 40 group: * *p* < 0.05; (**e**) centrifugal speeds, compared with 6000 group: * *p* < 0.05 ; (**f**) ultrasonic power, compared with 500 group: * *p* < 0.05.

In order to select the optimal concentration of ILs, a series of different concentrations of ILs was used. Considering the results in Figure 5a, which manifested that with the concentration of ionic liquid increasing, the extraction rate of target analytes showed an obvious upward trend (*p* < 0.05). The highest extraction yield was obtained when the IL concentration was 0.8 mol/L. From 0.8 to 1 mol/L, the extraction rate decreased obviously (*p* < 0.05). It is indicated that when the concentration of ILs increased, the extracting agent was hard to sufficiently contact with plant particles, so the ingredients could not be fully extracted from the medicinal herbs. Thus, the optimum ILs concentration was 0.8 mol/L. 

#### 2.4.2. Selection of Solid–Liquid Ratio

When the solid–liquid ratio was 1:20, the maximum extraction rate was reached. Experiments were carried out with a gradient of liquid–solid ratios (10, 20, 40, 60, 80, and 100 mL/g). In Figure 5b, the extraction yields increased significantly from 20 to 40 mL/g, and when the ratio was 40 mL/g, the extraction rate of four compounds reached the highest, and then the extraction rate decreased with the continuous increasing of the liquid–solid ratios. This may be related to the physical properties of ionic liquid itself. The results affirmed that the liquid–solid ratio of 20 mL/g was optimal for the following experiment.

#### 2.4.3. Selection of Mesh Number

Mesh number is one of the important factors that could significant influence the extraction rate of target analytes. As shown in Figure 5c, the extraction yields were increased with the increasing of mesh number, and the extraction rate of target analytes reached the maximum at 60 meshes, which was higher than others (*p* < 0.05). From 60 to 100 meshes, the extraction rate gradually decreased. The results indicated that ionic liquid may have some viscosity, which could lead to forming a cluster of the smaller particle sample, hindering the extraction effect [31]. Therefore, 60 meshes were selected for the subsequent experiment. 

#### 2.4.4. Selection of Ultrasonic Time

The data are shown in Figure 5d. In order to select the optimal ultrasonic time, ultrasonic times of 10, 20, 30, 40, 50, and 60 min were designed. The extraction rate of the target analytes basically showed a growth trend with the extension of the ultrasonic time. The extraction rate of the target analytes reached the maximum when the ultrasonic time was 40 min. However, from 40 to 60 min, the extraction rate displayed a downward trend. This may be because of the long ultrasonic time, which would destroy the structure of ILs [26,27]. Therefore, the optimal ultrasonic time was 40 min.

#### 2.4.5. Selection of Centrifugal Speed

Six different centrifugal speeds (3000, 4000, 5000, 6000, 7000, and 8000 r min^−1^) were designed to evaluate the effect of centrifugal speed on the extraction yield. The results are shown in Figure 5e. The extraction rate achieved the maximum at 6000 r min^−1^, which was markedly higher than others (*p* < 0.05). Thus, the 4000 r/min was selected as the optimal centrifugal speed for the experiments.

#### 2.4.6. Selection of Ultrasonic Power

The selection of ultrasound power was crucial for the experiment. In order to select the best ultrasound power, the effects of ultrasound power (100, 200, 300, and 500 W) were investigated. The effect of different ultrasonic power on extraction rate was regular. In Figure 5f, with the enhancement of the ultrasonic power, the extraction rate of the target analytes gradually increased. When the ultrasonic power was 500 W, the extraction rate of the target analytes reached the maximum, which was significantly higher than others (*p* < 0.05). It is probably because the plant tissues could be further broken by the shear force, which could significantly increase the permeability of solvent into plant cells and enhance the mass transfer rate [32]. Therefore, 500 W was selected for the subsequent single factor experiment.

### 2.5. Using Response Surface Method (RSM) to Optimize the Experimental Design

It is generally known that the extraction yield of target analytes is affected by multiple parameters, including the interactions of various factors on the extraction process. On the basis of various single factor assay, four single factors (concentration of ILs, solid-liquid ratio, centrifugal speed, and crushed mesh) were selected. To ascertain the optimum conditions for IL-UAE, the Box–Benhnken experiment designed with four-factors-three-levels was carried, using Design-Expert (DE) 8.0 software (Statease Inc., Minneapolis, MN, USA). Experimental factors and level design are shown in Table 1. In order to determine the impact of various factors and their interactions on the extraction process, the data in Table 1 were fitted with multiple regression using Design Expert 8.06 software to obtain the following quadratic multiple regression equation: *Y* = 281.60 − 2.19A − 21.02B − 6.35C − 5.66D + 6.79AB + 0.20AC + 17.96AD − 3.45BC − 6.95BD − 0.40CD − 46.44A^2^ − 29.16B^2^ − 32.34C^2^ − 13.56D^2^. In the equation, *Y* is the extraction yield of the target analytes from leaves of *S. octophylla*; and A, B, C and D are the concentration of ILs (A), solid–liquid ratio (B), centrifugal speed (C), and crushed mesh (D), respectively.

The analysis of variance for the fitted quadratic polynomial model for optimization of extraction parameters is presented in Table 2. The *p*-value for the model is *p* < 0.001, which indicated that the model was feasible. With very small *p*-values (*p* < 0.05), the results revealed that B, A^2^, B^2^, and C^2^ were significantly correlated with the yield of target analytes. The “lack of fit” was not significant relative to the pure error (*p* > 0.05), and adeq precision was 7.258, greater than 4, suggesting that this model could be used to predict the outcome of the experiment. There is a 5.63% chance that a “lack of fit F-value” this large could occur as a result of noise. 

**Figure 6:**
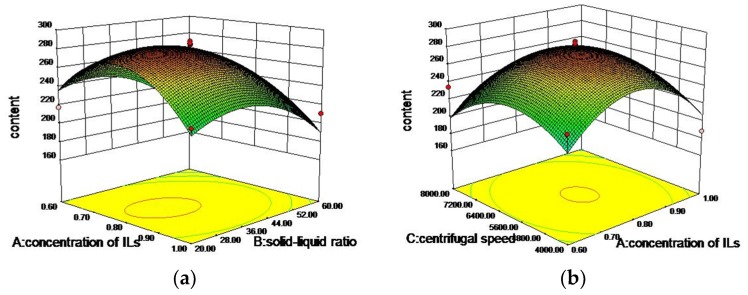

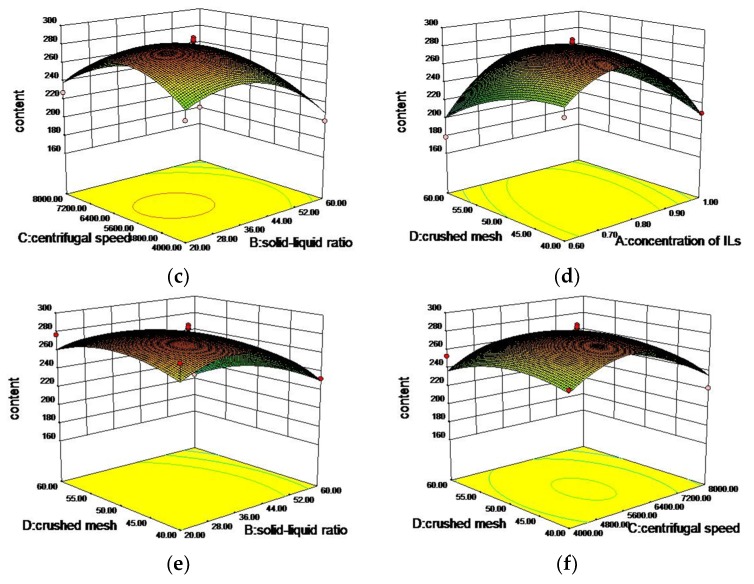
Three-dimensional (3D) response surfaces showing the effects of variables on extraction yield of target analytes. (**a**) Interaction of concentration of ILs and solid–liquid ratio; (**b**) interaction of concentration of ILs and centrifugal speed; (**c**) interaction of solid–liquid ratio and centrifugal speed; (**d**) interaction of crushed mesh and concentration of ILs; (**e**) interaction of crushed mesh and solid–liquid ratio; (**f**) interaction of crushed mesh and centrifugal speed.

The 3D response surfaces are shown in Figure 6, which shows the interactions of the four independent variables and the effect on extraction yield. In Figure 6a,b,d, the interactions between the concentration of ILs and the solid–liquid ratio, centrifugal speed, and mesh number had significant effects on the extraction rate of target analytes. When the other factors were determined, with the increasing of the concentration of ILs, the extraction rate of the target analytes first increased and then decreased, and vice versa. This could be explained in that the concentration of ILs increased the viscosity of ILs solutions [31], which affected the permeability of the ILs solution and the formation of mass transfer. In Figure 6c, from the sharpness of the response surface, we could see that the interaction between centrifugal speed and the solid–liquid ratio was significant. As we can see in Figure 6e,f, the interactions between the mesh number and the solid–liquid ratio and centrifugal speed had little effects on the extraction rate of target analytes. According to the response surface analysis, the optimal conditions of the whole experiment were determined as follows: the mesh number was 48.26, the concentration of ILs was 0.78 mol/L, the solid–liquid ratio was 1:33.11, and the centrifugal speed was 5839.93 r/min. In light of the actual situation, the optimal conditions were changed as follows: the mesh number was 50, the concentration of ILs was 0.8 mol/L, the solid–liquid ratio was 1:30, and the centrifugal speed was 6000 r/min. Under the optimal conditions, the extraction yield of target analytes from leaves of *S. octophylla* was 288.03 mg/g. It was close to the prediction value. Thus, the response surface method (RSM) was accurate and reliable.

### 2.6. Comparison with Conventional Solvents Extraction

In this study, we established the ILs-UAE method, and then selected and established the optimal extraction conditions of various single factors through response surface method (RSM). Under the optimal conditions, the content and extraction rate of *S. octophylla*, using 70% EtOH, were 220 ± 1.75 mg/g and 22%, respectively. [BMIM]BF4/70%EtOH was used as the solvent, and the content and extraction rate of *S. octophylla* could reach 288.03 ± 1.78 mg/g and 28.80%, respectively, which was 6.80% higher than that of 70% EtOH. It became clear from the results that the extraction rate of [BMIM]BF_4_/70%EtOH improved significantly when compared with that of 70% EtOH.

### 2.7. Method Validation

To develop the standard curve of target analytes, five different concentration levels of the standard solutions were analyzed. The regression equations, correlation coefficients, and linear ranges are listed in Table 3. The linearity was considerably good, with a regression coefficient greater than 0.999 and 0.994. In order to evaluate the experiment method of IL-UAE, four parameters (reproducibility, precision, stability, and recovery) were determined under the optimized conditions. The reduced swing differential signal (RSDs) values of target analytes were 0.49% and 0.73%, which indicated that the precision was acceptable and the instrument accurately measured the amount of the target analytes. The evidence of 1.74% and 1.11% from the RSDs of the method stated clearly that such an approach had good reproducibility and was reliable. The recovery rates of target analytes from dried leaves of *S. octophylla* were 101.69% and 98.47%, respectively. The RSDs values were 1.88% and 0.92%. The RSDs were 1.69% and 0.63%, with the time at 1, 3, 6, 9, 12, 20, and 24 h, respectively. The results demonstrated that the sample solution was relatively stable at room temperature within 24 h.

## 3. Materials and Methods

### 3.1. Chemicals and Materials

Methanol and acetonitrile were purchased from Tianjin Damao Chemical Reagent Factory (Tianjin, China). Ethyl acetate (EAC) was purchased from Tianjin Fuyu Fine Chemical Co. Ltd. (Tianjin, China). Water was purchased from Hangzhou Wahaha Baili Food Co. Ltd. (Hangzhou, China). Phosphocid was obtained from Tianjin Kermel Chemical Reagent Factory (Tianjin, China). 1-Butyl-3-methylimidazolium tetrafluoroborate ([BMIM]BF_4_), 1-butyl-3-methylimidazolium bromide ([BMIM]Br), and 1-butyl-3-methylimidazolium hexafluorophosphate ([BMIM] PF_6_) were obtained from limited partnership Merck (Darmstadt, German). 1-Hexyl-3-methylimidazolium hexafluorophosphate ([HMIM]PF_6_) was purchased from Thermo Fisher Scientific (Rock-ville, MD, USA). Dulbecco’s modified Eagle’s medium (DMEM), neutral red was purchased from Solarbio (Beijing, China). Fetal bovine serum (FBS) was purchased from Gibco (Grand Island, NY, USA). Nitric oxide kit was purchased from Nanjing Jancheng Bioengineering Institute (NanJing, China) Lipopolysaccharide (LPS) was purchased from Sigma (St. Louis, MO, USA). Compounds **A** and **B** (3α-hydroxy-lup-20(29)-ene-23,28-dioic acid and 3α-hydroxy-lup-20(29)-ene-23,28-dioic acid 28-*O*-[α-l-rhamnopyranosyl(1→4)-*O*-β-d-glucopyranosyl(1→6)]-β-d-glucopyranoside) with purity >98% were isolated from the leaves of *S. octophylla*.

An LC-20AT high performance liquid chromatography system (Shimadzu, Kyoto, Japan) equipped with a degasser, a quaternary gradient low pressure pump, the CTO-20A column oven, a SPD-M20AUV-detector, and an SIL-20A auto sampler was used. Chromatographic separations were performed on an Agilent ZORBAX SB-C18 column (4.6 mm × 5 mm, 5μm) and KQ-500 dB ultrasonic cleaner (Jiangsu Kunshan Ul-trasonic Instrument Co., Ltd. Kunshan, China). TGL-16 type high speed centrifuge was obtained from Jiangsu Jintan Zhongda instrument factory (Jintan, China). AB135-S 1/10 million electronic balance was purchased from Mettler Toledo Instruments Co., Ltd. (Shanghai, China).

### 3.2. Plant Materials

*Schefflera octophylla* (Lour.) Harms (*S. octophylla*) fresh leaves were collected from Quanzhou (118°36’ E, 24°58’ N), Fujian Province, China, in April 2017, and the plant was identified by Prof. C. Q. Li (Henan University, Kaifeng, China). A voucher specimen (No. 201704021) was deposited in Henan University. 

### 3.3. Cell Culture and Treatment

RAW264.7 macrophages were purchased from Shanghai Institutes for Biological Sciences (Chinese Academy of Sciences, Shanghai, China). The cells were cultured in DMEM (Dulbecco’s modified Eagle’s medium) with 10% FBS (fetal calf serum), 100 U/mL penicillin, and 100 µg/mL of streptomycin. The experimental group treated with different concentrations of compound **A** and compound **B**. The blank control group was culture medium and the positive control group was culture medium + LPS (1 µg/mL).

### 3.4. Cell Viability Assay

RAW264.7 cells were cultured in 96-well plates (1 × 10^5^ cells/mL) and incubated at 37 °C and 5% CO 2 for 24 h. The cells were treated with various concentrations of compound **A** and compound **B**. When 10 µL of MTT solution was added to each well, succinate dehydrogenase in mitochondria of living cells was reduced to water-insoluble blue-purple crystallize, which had the best absorption at 490 nm.

### 3.5. Nitric Oxide (NO) Assay

RAW 264.7 cells were cultured in 24-well plates (1×10^6^ cells/mL) and treated with different concentrations of compound **A** and compound **B** for 24 h. The supernatant was collected to determine the levels of NO using the nitric oxide kit.

### 3.6. Preparation of the Standard Solution and Test Sample Solution

Certain amounts of compound **A** and compound **B** were weighed and dissolved with methanol, at a concentration of 7.068 mg/mL and 0.49 mg/mL, which was mixed as the standard solution.

Then, 50 mg of leaves of *S. octophylla* was weighed and dissolved in 1 mL of extraction solution (70% EtOH and ILs), after which the sample was ultrasonic treated for 30 min at room temperature and centrifuged for supernatant, which was filtered by a 0.22 μm organic microporous membrane to obtain the sample solution. In the experiment, variables of ILs types, the concentration ILs, particle size, sample–solvent ratio, ultrasonic time, ultrasonic power, and centrifugal speed were evaluated in turn. Each test was performed in parallel in triplicates. 

### 3.7. Graphic Conditions

**Figure 7:**
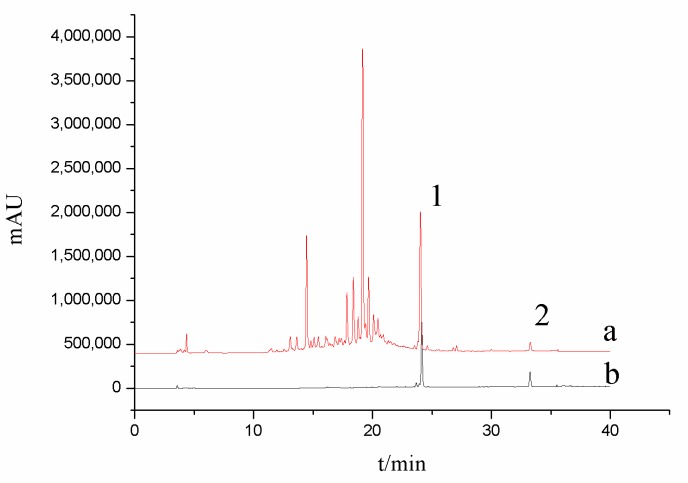
High performance liquid chromatography (HPLC) chromatograms of the test sample solution (a) and the standard solution (b): 1. Compound **B**, 2. Compound **A**.

The HPLC conditions are shown in Table 4. Under this chromatographic condition, The HPLC chromatograms of the standard solution and the sample solution are shown in Figure 7. The peak retention time of compound **A** and compound **B** in the sample was in accordance with the peak retention time of mixed standard. The separation degree of compound **A** and compound **B** was good in the sample, at 0.752 and 1.015, respectively. 

### 3.8. Statistical Analysis

All the experimental data were expressed as mean ± standard deviation (SD). Statistical analysis was performed with the SPSS19.0. Comparison between any two groups was evaluated using one-way analysis of variance (ANOVA). Design Expert 8.0 (DE, Statease Inc., Minneapolis, MN, USA) was used to analyze the experimental data and obtain the response models. 

## 4. Conclusions

To the best of our knowledge, this was the first time that investigators used the method of ionic liquid-ultrasonic assisted extraction and to measure the extraction yields of two main anti-inflammatory triterpenoids in leaves of *S. octophylla*. The optimal conditions were set up as follows: 50 mesh powders, concentration of ILs was 0.8 mol/L, the ultrasonic time was 40 min, the solid–liquid ratio was 1:30, and centrifugal speed was 6000 r/min. Under these conditions, the extraction rate of compound **A** and compound **B** in leaves of *S. octophylla* was increased by 6.80% compared with the traditional methods.

## Figures and Tables

**Table 1:** Box–Behnken design (BBD) for the independent variables and corresponding response values. ILs, ionic liquids.

Independent Variable	Level		
	−1	0	1
ILs concentration (mol/L)	0.6	0.8	1.0
Solid–liquid ratio (g/mL)	1:20	1:40	1:60
Centrifugal speed (r/min)	4000	6000	8000
Mesh number (mesh)	40	50	60

**Table 2:** Analysis of variance (ANOVA) for the fitted quadratic polynomial model for optimization of extraction parameters.

Source	Sum of Squares	df	Mean Square	F Value	*p*-Value Prob. F	
Model	27581.58	14	1970.11	5.24	0.0019	**
A	57.51	1	57.51	0.15	0.7016	
B	5301.24	1	5301.24	14.10	0.0021	**
C	483.24	1	483.24	1.29	0.2760	
D	383.75	1	383.75	1.02	0.3295	
AB	184.28	1	184.28	0.49	0.4953	
AC	0.16	1	0.16	4.255 × 10^−4^	0.9838	
AD	1290.25	1	1290.25	3.43	0.0852	
BC	47.61	1	47.61	0.13	0.7273	
BD	193.071	1	1193.07	0.51	0.4854	
CD	0.63	1	0.63	1.681 × 10^−3^	0.9679	
A^2^	13986.72	1	13986.72	37.20	<0.0001	***
B^2^	5516.28	1	5516.28	14.67	0.0018	**
C^2^	6785.46	1	6785.46	18.05	0.0008	***
D^2^	1193.50	1	1193.50	3.17	0.0965	
Residual	5263.98	14	376.00			
Lack of fit	4910.78	10	491.08	5.56	0.0563	No significant
Pure error	353.20	4	88.30			
Cor total	32845.58	28				

*** Extremely significant (*p* < 0.001). ** Highly significant (*p* < 0.01).

**Table 3:** Regression equations, linear ranges, and correlation coefficients.

NO.	Regression Equation	Linear Range (μg/mL)	Correlation Coefficients
Compound **A**	*Y* = 336721 X+112706	0.98~14.7	R^2^ = 0.9997
Compound **B**	*Y* = 134770 X+2456878	14.136~212.04	R^2^ = 0.9948

**Table 4:** Chromatographic conditions.

Chromatographic Conditions	Parameter
Column	Thermo ODS-2 HYPERSIL column (4.6 mm × 250 mm, 5 μm)
Mobile phase	Acetonitrile (A)0.1% phosphoric acid aqueous solution (B) gradient elution: 0–40 min 5%~100% A 95%~0% B
Flow rate	0.8 mL·min^−1^
Column temperature	25 °C
Wavelength	210 nm
Sample volume	10 μL
